# Floquet angular modulation for 6G systems

**DOI:** 10.1038/s41598-026-42429-8

**Published:** 2026-03-03

**Authors:** Bilel Hamdi, Radhoine Aloui, Adel Sharar Aldalbahi, Tijeni Delleji, Sofien Mhatli, Taoufik Aguili, Ignacio Llamas-Garro, Ahmed Siala

**Affiliations:** 1 Laboratory of Communication Systems Sys’Com (LR-99-ES21), National Engineering School of Tunis, University of Tunis El Manar, BP.37 Le Belvédère, Tunis, 1002 Tunisia; 2 Science and Technology for Defense Lab (STD), Military Research Center, Aouina Military Base Taieb Mhiri City, 2045 Tunis, Tunisia; 3 Department of Electrical Engineering, College of Engineering, King Faisal University, 31982 Al-Ahsa, Saudi Arabia; 4 SERCOM-Lab (LR13ES09), Tunis Polytechnic School, University of Carthage, Khawarizmi Street, P.O. Box 743, 2078 Al Marsa, Tunis, Tunisia; 5 Centre Tecnologic de Telecomunicacions de Catalunya (CTTC/CERCA), Av. Carl Friedrich Gauss 7, Castelldefels, 08860 Barcelona Spain; 6 Higher Institute of Computer Science of Le Kef (ISI Kef), University of Jendouba, 7100 Le Kef, Tunisia

**Keywords:** Engineering, Mathematics and computing, Optics and photonics, Physics

## Abstract

This paper introduces a computationally efficient method for angular modulation analysis in 5G/6G systems, significantly reducing numerical complexity and computation time compared to conventional techniques. Inspired by optical spectroscopy, we present novel approaches tailored for 5G/6G applications like reconfigurable intelligent surfaces (RIS) and metasurfaces. Unlike traditional direct methods (Fourier, Jones matrix, Bessel series, orbital angular momentum), our technique employs a modified Fourier method combined with Floquet analysis.

## Introduction

The paradigm shift toward sixth-generation (6G) wireless networks necessitates revolutionary advances in three core domains: ultra-high spectral efficiency, sub-degree beamforming precision, and real-time adaptive wavefront control^33–38^. These capabilities are critical for emerging applications including holographic communications, pervasive sensing, and terahertz (THz)-band connectivity^36,37^. Central to this evolution are *reconfigurable intelligent surfaces* (RIS) and electromagnetic metasurfaces^38^, which enable unprecedented spatio-temporal manipulation of electromagnetic fields through angular modulation–a fundamental technique governing phase-front engineering for beam steering^14^, orbital angular momentum (OAM) multiplexing^33–35^, and interference nulling^35^. Traditional analytical models for angular modulation face significant limitations when applied to dynamic 6G scenarios:

- Fourier analysis^1–11^ assumes time-invariant systems, struggling with rapidly varying phase responses in reconfigurable metasurfaces.
- Jones matrix formalism^12–18^, while powerful for polarization control, lacks native support for spectral harmonic interactions in periodic structures.
- Bessel series decompositions^18–32^ become computationally intractable for arbitrary nonlinear modulations.
- OAM-based methods^33–35^ exhibit sensitivity to misalignment and spatial perturbations.

To contextualize our proposed Fourier-Floquet analysis, we first survey established angular modulation techniques and their limitations for 6G systems. Section 2 provides a comprehensive review of Fourier analysis^1–11^, Jones matrix formalism^12–18^, Phase Modulation Spectroscopy^19–32^, Orbital Angular Momentum^33–35^, and other relevant methods^36–40^. Inspired by precision spectroscopic techniques in photonics^23–32,31,53,57^, this article introduces a modified Fourier-Floquet analysis as a unified model for angular modulation in 5G/6G systems. Our approach synergistically combines^1,5–7,9^:

$$\begin{aligned} & \underbrace{Floquet-Bloch -decomposition}_{\text {Periodic -structure- analysis}} \\ & \quad +\underbrace{Generalized-angular-modulation}_{\text {Arbitrary -phase- control}} \\ & \quad \rightarrow Spectral-spatial-co-design \end{aligned}$$

Unlike conventional methods, we use *Floquet’s theorem*^48,49^ to decompose wave propagation in periodic RIS arrays into spatial harmonics, enabling:

- Efficient unit cell reduction for infinite arrays via phase-shift boundary conditions $e^{jk_x d}$^48,49^.
- Spectral convolution formalism for nonlinear modulation effects^31^ :
  $$\begin{aligned} u_{\text {mod}}(x) = \sum _p (\hat{\phi } *c)_p e^{i\left( k + p\frac{2\pi }{d}\right) x}, \quad (\hat{\phi } *c)_p = \sum _n \hat{\phi }_n c_{p-n} \end{aligned}$$
- Time-domain extensibility for Floquet-engineered beam steering^18–32^: $\theta (x,t) = \beta x + \Delta \theta \cos (\Omega t)$^11^.

This initiative advances the state-of-the-art in three key domains:

1. RIS beamforming: Derives generalized Snell’s law for programmable phase gradients^36^: $\sin \theta _r = \sin \theta _i + \frac{\beta \lambda }{2\pi }$.
2. Multi-beam generation: Enables *N*-beam synthesis via nonlinear modulation spectra $c_m$^31^.
3. MIMO-RIS integration: Models channel matrix as $\textbf{H}$ = $\sum _n \textbf{H}_n e^{i n \frac{2\pi }{d} x}$ for capacity optimization^33–36^.

The paper is structured as follows: Section 2 reviews angular modulation fundamentals. Section 3 details the Fourier-Floquet methodology. Section 4 applies this model to RIS-assisted 6G systems. Section 5 presents numerical validation. Conclusions outline future work. A summary of key mathematical notation is provided in Appendix D (Table 5) for reference.

## Background on angular modulation techniques

There are four main techniques that explain the concept of angular modulation based on reflective and absorptive surfaces for antenna arrays^1–36^:

### Fourier analysis

The papers^9–11^ analyze distortion in angular-modulated signals of the form

1 $$\begin{aligned} e(t) = E \sin [\omega _0 t + \theta (t)] \end{aligned}$$

when transmitted through a network with transfer function $Z(\omega )$. Using Fourier analysis^1–11^, the output signal is expressed as:

2 $$\begin{aligned} e_0(t) = \frac{1}{2\pi } \int _{-\infty }^{\infty } e(t-\tau ) \int _{-\infty }^{\infty } Z(\omega ) e^{i\omega \tau } d\omega d\tau \end{aligned}$$

Key findings demonstrate that:

- Linear-phase transfer characteristics ($Z(\omega ) = e^{i\beta \omega }$) preserve signal integrity, introducing only a time delay $\beta$:
  3 $$\begin{aligned} e_0(t) = e(t - \beta ) \end{aligned}$$
- Hyperbolic cosine amplitude characteristics ($|Z(\omega )| = \cosh \lambda (\omega -\omega _0)$) enhance high frequencies without distortion:
  4 $$\begin{aligned} e_0(t) = \frac{E}{2} e^{i\omega _0 t} \left[ e^{i\theta (t-\lambda )} + e^{i\theta (t+\lambda )} \right] \end{aligned}$$
- Sinusoidal amplitude characteristics ($|Z(\omega )| = \cos (\lambda \omega + \Omega )$) attenuate high frequencies, with maximum modulation frequency $f_s$ limited by:
  5 $$\begin{aligned} f_s \approx 0.9B\sqrt{\delta } \end{aligned}$$
  where $B$ is the 3dB bandwidth and $\delta$ the tolerable signal depression.

The analysis concludes that phase linearity is more critical than amplitude flatness for distortion minimization in angular modulation systems^1–11^.

### Jones matrix formalism

The Jones matrix formalism models the transformation of polarized light by optical elements using linear algebra^12–18^. The polarization state of a monochromatic plane wave is represented by a Jones vector $$\textbf{E} = \begin{bmatrix} E_x \\ E_y \end{bmatrix}$$, where $E_x$ and $E_y$ are complex amplitudes describing the electric field components along the $x$- and $y$-axes. An optical element (e.g., polarizer, waveplate) is characterized by a $2 \times 2$ complex Jones matrix $$\textbf{J} = \begin{bmatrix} J_{xx} & J_{xy} \\ J_{yx} & J_{yy} \end{bmatrix}$$. The output polarization state after interaction is computed as $\textbf{E}_{\text {out}} = \textbf{J} \textbf{E}_{\text {in}}$. For a system of $N$ sequential elements, the total effect is given by the product of their individual matrices: $\textbf{E}_{\text {out}} = \textbf{J}_N \cdots \textbf{J}_1 \textbf{E}_{\text {in}}$. Common matrices include a linear polarizer aligned to the $x$-axis, $$\begin{bmatrix} 1 & 0 \\ 0 & 0 \end{bmatrix}$$, and a quarter-wave plate with its fast axis along $x$, $$\begin{bmatrix} 1 & 0 \\ 0 & i \end{bmatrix}$$. Rotated elements are described using a rotation matrix $$\textbf{R}(\theta ) = \begin{bmatrix} \cos \theta & -\sin \theta \\ \sin \theta & \cos \theta \end{bmatrix}$$, with the transformed Jones matrix $\textbf{J}' = \textbf{R}(-\theta ) \textbf{J} \textbf{R}(\theta )$. The intensity of the output is $I = \textbf{E}_{\text {out}}^\dagger \textbf{E}_{\text {out}}$, where $\dagger$ denotes the conjugate transpose. This model applies to coherent, non-depolarizing systems.

The Jones matrix formalism, a powerful tool for modeling polarization states in optical systems, can be adapted for 6G communication systems to analyze and optimize polarization-sensitive components, such as reconfigurable intelligent surfaces (RIS) and multi-antenna arrays^12–18^. By leveraging Floquet angular modulation–a periodic modulation technique inspired by Floquet theory–researchers can enhance signal robustness and spectral efficiency in 6G networks. This approach enables precise control over wavefronts and polarization states, mitigating interference and improving channel capacity. The combination of Jones matrix analysis and Floquet modulation offers a promising model for advancing next-generation wireless systems, particularly in scenarios requiring high-frequency terahertz (THz).

### Phase modulation spectroscopy (PMS)

Employs sinusoidal phase modulation of a laser beam^12–32^:

$$\begin{aligned} E(t)&= E_0 \exp [i(\omega _0 t + M \sin \omega _m t)] \\&= E_0 \exp (i\omega _0 t) \sum _{n=-\infty }^{\infty } J_n(M) \exp (i n \omega _m t) \end{aligned}$$

where $M$ = modulation index, $\omega _m$ = modulation frequency, $J_n$ = Bessel functions. After sample transmission (absorption $\delta (\omega )$, dispersion $\phi (\omega )$), the $\omega _m$-signal contains:

- Absorption component: $$\propto \displaystyle \sum _{n=0}^{\infty } J_n J_{n+1} (\delta _{-n-1} - \delta _{n+1} + \delta _{-n} - \delta _n) \cos \omega _m t$$
- Dispersion component: $$\propto \displaystyle \sum _{n=0}^{\infty } J_n J_{n+1} (\phi _{-n-1} - \phi _{-n} + \phi _{n+1} - \phi _n) \sin \omega _m t$$

### Angular modulation (FM/WM spectroscopy)

Defined via instantaneous frequency:

$$\omega _i(t) = \omega _0 + \Delta F \cos \omega _m t, \quad \Delta F = M \omega _m$$

- Frequency Modulation (FM): $M \ll 1$, $\omega _m \gg \Gamma$ (linewidth). Signal from one sideband:
  $$I \propto M(\delta _{-1} - \delta _1) \quad \text {(absorption)},$$
  $$\quad I_k \propto -\delta _k \frac{4k}{M} J_k^2(M) \quad (M \gg 1)$$
- Wavelength Modulation (WM): $M \gg 1$, $\omega _m \ll \Gamma$. Approximates absorption derivative:
  $$I_{\text {WM}} \propto -\frac{da}{d\omega } \Delta F, \quad a = 2\delta \quad \text {(intensity absorption)}$$

Note: $\delta$ = field attenuation, $a$ = intensity absorption ($a = 2\delta$), $\Gamma$ = spectral linewidth. FM/WM unified under angular modulation in $(M, \omega _m)$ parameter space.

### Orbital angular momentum (OAM)

is a property of electromagnetic waves characterized by helical wavefronts, offering an additional degree of freedom for spatial multiplexing in 6G communications. The complex amplitude of an OAM mode of order $\ell$ is expressed as:

$$\psi (r,\phi ,z) \propto e^{i\ell \phi }$$

where $\ell$ is the azimuthal quantum number (integer value). In 6G waveform design, OAM enables an increase in spectral capacity through multiplexing of orthogonal modes:

$$\text {Capacity} \propto \sum _{\ell =-\infty }^{\infty } \log _2(1 + \textsf{SNR}_\ell )$$

Floquet angular modulation exploits the temporal dynamics of OAM vortices to encode information, optimizing robustness in multipath channels. Key performances:

- Spectral density: $\eta \approx \ell _{\text {max}} \times \eta _0$ ($\ell _{\text {max}}$: maximum usable modes).
- Error rate: $\text {SER} \propto e^{-\gamma \cdot \Delta \ell ^2}$ ($\Delta \ell$: mode separation).

6G Advantages: Reduction of inter-mode interference and scalability for THz, subject to beam divergence and alignment constraints^33–35^.

### Other kinds of angle modulation applications

The articles^36,37^ describe a geometrical phase shift keying (PSK) modulation technique using a graphene-based reflectarray metasurface. By spatially shifting a periodic control pattern (e.g., alternating ON/OFF states of cells) across the array, the phase of the reflected terahertz beam is modulated. Each shift by one cell introduces a precise phase shift of $2\pi /P$ (where P is the pattern period), enabling broadband phase modulation without altering the beam’s frequency. This approach leverages the reconfigurable phase hologram of the reflectarray to encode information or steer beams, with applications in terahertz communications and adaptive optics.

The paper^39^ employs linear frequency modulation (LFM) of an optical carrier (laser) for applications such as FMCW lidar. By linearly sweeping the laser frequency over a bandwidth $\Delta f$, the distance to a target is determined from the beat frequency between the transmitted and reflected waves. This angle modulation technique, implemented via electro-optic or acousto-optic modulators, offers advantages including noise immunity, high linearity, and precise ranging capabilities in space communication and sensing.

Angular modulation refers to designing an optical element (here, a metasurface) with a phase profile that deliberately varies with the azimuthal angle ($\theta$), breaking rotational symmetry. By incorporating a term proportional to $\theta \cdot r^4$ in the phase equation (Eq. 2), it adds an extra degree of freedom beyond radial position. This enables an extended focal segment (long DOF) while maintaining image quality over the range, overcoming limitations of rotationally symmetric elements like axicons. The approach is particularly powerful in metasurfaces, allowing ultrathin, polarization-insensitive, and broadband extended-DOF devices^38^.

Beyond extended depth of focus, metasurfaces have also been leveraged for generating orbital angular momentum (OAM) beams with high efficiency and purity. Recent advances include dielectric metasurfaces that convert linearly polarized light into high-purity OAM modes with topological charges up to $\pm 10$^67^, and geometric-phase metasurfaces that enable broadband OAM multiplexing across a wide spectral range^68^. These demonstrations highlight the potential of metasurfaces as compact and efficient OAM generators, which can be further optimized using the Floquet-based angular modulation model proposed in this work. The paper^40^ employs polarization modulation of IR light (switching between *s*- and *p*-polarizations) to simultaneously probe surface-adsorbed species and liquid-phase species at catalytic solid-liquid interfaces. This enables time-resolved monitoring of reactions (e.g., CO oxidation on Pt) by selectively isolating surface and bulk signals. The paper^13^ uses polarization modulation via a rotating quarter-wave plate to process orthogonally circularly polarized radio beacon signals. The bearing (direction) of a moving object is determined from the phase of the 4th harmonic ($4\Omega$) of the modulation frequency, leveraging the relationship $\varphi _{4\Omega } = \Delta \varphi$ for navigation.

Both papers utilize polarization modulation as a form of angle modulation—^40^ for spectroscopic interfacial analysis and^13^ for navigation bearing determination—demonstrating its versatility in extracting phase-sensitive information.

### Current methods for RIS and OAM: Merits and Limitations

The diverse angle modulation techniques outlined in Sections 1.1−1.5 form the foundational toolkit for wavefront engineering. However, when applied to the core 6G technologies of Reconfigurable Intelligent Surfaces (RIS) and Orbital Angular Momentum (OAM) communications, each method reveals distinct merits and practical limitations in dynamic, large-scale scenarios. This subsection provides a focused comparison of these approaches, highlighting their computational trade-offs and scalability challenges for next-generation systems.

#### RIS methods

- **Fourier-Based Phase Retrieval** Fourier methods are widely used for phase profile synthesis in RIS due to their simplicity and speed in the spectral domain. They allow efficient analysis of periodic or quasi-periodic structures via Floquet harmonics. However, these methods assume time-invariant systems and struggle with rapid reconfiguration, nonlinear phase responses, and edge effects in finite arrays, limiting their applicability in real-time adaptive RIS^1–11,46–48^.
- **Jones Matrix Modeling** Adapted from polarization optics, Jones matrices provide a powerful framework for modeling polarization-sensitive RIS elements and multi-antenna arrays. They enable precise control over wavefront polarization states, which is critical for polarization multiplexing and interference mitigation. Nonetheless, the formalism lacks native support for spectral harmonic interactions and nonlinear modulation effects, making it less suitable for broadband or dynamically tuned metasurfaces^12–18^.
- **Bessel Series Decomposition** Bessel expansions are commonly employed to analyze sinusoidal or periodic phase modulations, such as those used in Phase Modulation Spectroscopy (PMS). While accurate for certain modulation profiles, Bessel series become computationally prohibitive for arbitrary or nonlinear phase functions, with complexity scaling as $\mathscr {O}(N^2)$ for large arrays. This limits their use in real-time optimization of reconfigurable metasurfaces^19–32^.
- **Computational Challenges in Dynamic 6G Environments** In 6G scenarios requiring sub-millisecond reconfiguration, high-dimensional optimization, and integration with massive MIMO, traditional RIS methods face significant bottlenecks. Finite array effects, manufacturing tolerances, and element non-uniformity further degrade performance, necessitating robust and efficient modeling frameworks such as the Floquet-based approach proposed in this work^63–65^.

#### OAM methods

- **Mode Multiplexing** OAM mode multiplexing exploits orthogonal helical wavefronts to increase spectral efficiency, offering a theoretically unbounded number of channels. It is particularly promising for line-of-sight THz communications. However, OAM modes are highly sensitive to misalignment, atmospheric turbulence, and multipath scattering, which can lead to severe inter-mode crosstalk and limit practical deployment^33–35^.
- **Holographic Metasurfaces** Metasurface-based OAM generators use holographic principles to imprint phase profiles that produce OAM beams. These devices are compact, integrable, and capable of multi-mode generation. Nevertheless, they suffer from limited bandwidth, polarization sensitivity, and design complexity, especially when targeting high-order OAM modes at THz frequencies^36–38^.
- **Spatial Phase Modulation** Spatial light modulators (SLMs) and programmable metasurfaces can dynamically generate OAM beams via real-time phase modulation. While flexible, these systems require high-resolution control, exhibit diffraction losses, and face scalability issues in large-aperture or high-frequency implementations^38,39^.
- **Scalability and THz Band Limitations** At THz frequencies, OAM systems encounter challenges such as beam divergence, atmospheric absorption, and fabrication precision. The number of usable OAM modes is often limited by aperture size and alignment stability, constraining the practical capacity gains in 6G links^34,35^.

Recent surveys on RIS and OAM techniques further underscore these trade-offs, emphasizing the need for hybrid analytical-computational frameworks that combine accuracy, efficiency, and adaptability for 6G systems^33–38,59–62^.

## Angle-modulated floquet analysis (a new modified technique)

### Fourier-Floquet analysis

The Fourier-Floquet analysis provides a powerful model for characterizing wave propagation in periodic antenna arrays by decomposing the problem into fundamental spatial harmonics. This analysis is based on the assumption of an infinitely extended periodic structure, which allows the application of Floquet’s theorem and reduces the problem to the study of a single unit cell with phase-shift boundary conditions [49, 50]. This approach—which we have previously applied to model finite and infinite planar structures [49, 65]—simplifies the analysis of an infinite periodic array by reducing it to the study of a single unit cell through the application of Floquet’s theorem, which introduces a phase shift boundary condition to account for periodicity [49]. The key insight is that the array’s transfer function can be completely described by analyzing one unit cell while incorporating the Floquet phase shift $e^{jk_xd}$ between adjacent cells, where $k_x$ represents the wavevector component parallel to the periodicity direction and *d* is the spatial period^6,9^. The complete spatial response of the array is then obtained via superposition of all Floquet-Bloch modes in the spectral domain. As shown in Eq.2.7 of^44^, the dispersion relation $\omega (k_x)$ for Bloch waves exhibits periodicity in k-space with a period of $2\pi /d$, meaning the wave behavior repeats every reciprocal lattice vector $2\pi l/d$ (where $l = \pm 1, \pm 2,...$). This periodicity implies that wavevectors differing by integer multiples of $2\pi /d$ are physically equivalent - a fundamental consequence of the underlying spatial periodicity that leads to the folding of dispersion curves into the first Brillouin zone $[-\pi /d, \pi /d]$. Figures 2.10a and 2.10b of^44^ illustrate this critical difference between periodic and non-periodic media, where the periodic case shows band folding and the emergence of photonic band gaps due to constructive interference of scattered waves^44^. The Fourier-Floquet method thus provides both computational efficiency (through unit cell reduction) and physical insight into how periodic structuring modifies wave propagation.

#### Implications of the infinite-array assumption

The infinite-periodicity assumption yields two key implications:

1. **Computational Efficiency:** The reduction to a single unit cell with Floquet phase-shift conditions transforms the analysis into a spectral problem solvable via Fast Fourier Transform (FFT) algorithms. This reduces the computational complexity from $\mathscr {O}(N^2)$ (direct spatial methods) to $\mathscr {O}(N \log N)$, where *N* is the number of spatial harmonics. This efficiency enables real-time optimization of large-scale RIS and metasurface configurations.
2. **Neglect of Edge Effects:** The model does not account for truncation at array boundaries. For finite arrays—especially with $N < 100$ elements—edge effects can lead to beam squint, elevated side lobes, and pattern distortion. For large arrays ($N > 1000$), these effects become relatively less significant in the central region, making the infinite-array approximation a valid and efficient design tool. The proposed method is therefore particularly intended for large-scale RIS and metasurfaces where edge effects are relatively small, and serves as an efficient foundation for optimization. Mitigation strategies for finite arrays, such as windowing techniques (e.g., Hann, Hamming) or the array scanning method, are discussed in Section 5 and have been explored in our previous work [64] and in the literature [65, 66]. These approaches can be incorporated into the Floquet spectral model as straightforward extensions for handling practical finite-sized metasurfaces.

### Modified technique

Core Innovation: While Section 3-1 describes standard Floquet analysis for periodic structures, our modification introduces angular modulation $\theta (x)$ as an independent control dimension. The key advancement is treating $e^{i\theta (x)}$ as a separate spectral operator that convolves with the Floquet spectrum, enabling:

- Arbitrary wavefront engineering beyond structural periodicity
- Real-time reconfiguration without changing physical geometry
- Nonlinear modulation analysis via $c_m$ coefficients

This transforms Floquet theory from a passive analysis tool into an active design model.

To derive the equation transforming the Floquet spectral wavenumber domain to the space domain with angular modulation, we follow a rigorous mathematical approach. First, using the Floquet–Bloch theorem, a wave solution $u(x)$ in a periodic medium with period $d$ (the unit cell size) is expressed as a Bloch wave:

$$u(x) = e^{i k x} \phi (x),$$

where $k$ is the Floquet exponent (or Bloch wavenumber) and $\phi (x)$ is a periodic function with period $d$, i.e., $\phi (x+d) = \phi (x)$. Expanding $\phi (x)$ in a Fourier series gives

$$\phi (x) = \sum _{n=-\infty }^{\infty } \hat{\phi }_n e^{i n \frac{2\pi }{d} x},$$

where $\hat{\phi }_n$ are the Fourier coefficients of $\phi (x)$. This leads to

$$u(x) = \sum _{n=-\infty }^{\infty } \hat{\phi }_n e^{i \left( k + n \frac{2\pi }{d} \right) x}.$$

Introducing angular modulation via a phase function $\theta (x)$, which represents the desired phase profile, the modulated field becomes

$$u_{\text {mod}}(x) = e^{i \theta (x)} \sum _{n=-\infty }^{\infty } \hat{\phi }_n e^{i \left( k + n \frac{2\pi }{d} \right) x}.$$

For linear modulation ($\theta (x) = \beta x$), this simplifies to a shift in the Floquet exponent:

$$u_{\text {mod}}(x) = \sum _{n=-\infty }^{\infty } \hat{\phi }_n e^{i \left( k + \beta + n \frac{2\pi }{d} \right) x}.$$

The general form, accounting for arbitrary modulation, is

$$u_{\text {mod}}(x) = e^{i \theta (x)} \sum _{n=-\infty }^{\infty } \hat{\phi }_n e^{i \left( k + n \frac{2\pi }{d} \right) x},$$

representing the transformation from the Floquet spectral domain to the space domain with angular modulation^62^.

Alternative Form: Convolution in Spectral Domain

If the angular modulation $\theta (x)$ admits a Fourier expansion, we can write

$$e^{i \theta (x)} = \sum _{m=-\infty }^{\infty } c_m e^{i m \frac{2\pi }{d} x},$$

where $c_m$ are the Fourier coefficients of $e^{i\theta (x)}$. Then the modulated field can be expressed as a double summation:

$$u_{\text {mod}}(x) = \sum _{n=-\infty }^{\infty } \sum _{m=-\infty }^{\infty } \hat{\phi }_n c_m e^{i \left( k + (n + m) \frac{2\pi }{d} \right) x}.$$

This representation reveals a spectral convolution between the Floquet harmonics $\hat{\phi }_n$ and the modulation coefficients $c_m$. Physically, this means that each Floquet mode $n$ couples with every modulation harmonic $m$, producing shifted spectral components at $k + (n + m)\frac{2\pi }{d}$. Such a formulation is particularly useful for analyzing nonlinear interactions or arbitrary phase modulations in periodic systems.

Letting $p = n + m$, we can rewrite the above as

$$u_{\text {mod}}(x) = \sum _{p=-\infty }^{\infty } (\hat{\phi } * c)_p e^{i \left( k + p \frac{2\pi }{d} \right) x},$$

where $(\hat{\phi } * c)_p = \sum _{n=-\infty }^{\infty } \hat{\phi }_n c_{p-n}$ denotes the discrete convolution of the sequences $\hat{\phi }_n$ and $c_m$. A summary of key notation is provided in Appendix 5 (Table 5).

The proposed modified Fourier-Floquet analysis establishes a unified framework for modeling complex wavefront manipulation in reconfigurable metasurfaces. This approach integrates recent advances in Floquet engineering^49–62^ to address critical challenges in 6G systems, particularly for reconfigurable intelligent surfaces (RIS) and metasurface applications. By leveraging spectral convolution techniques within a Floquet-Bloch formalism, the method enables efficient analysis of arbitrary phase modulations while maintaining computational tractability for large-scale arrays.

## Floquet theory with angular modulation in 6G, reconfigurable intelligent surfaces (RIS) and meta-surfaces: mathematical formulation

Applying our modified Floquet analysis to 6G systems provides a powerful model for analyzing wave propagation in 6G communication systems and Reconfigurable Intelligent Surfaces (RIS)^1–62^. Below, we derive the key equations and discuss their applications.

### Floquet-Bloch modes in periodic RIS structures

Consider an RIS with a periodic unit cell of size $d$. The wave interaction can be modeled using Floquet-Bloch theory^45,48–51^:

#### Floquet-Bloch decomposition

The electric field $E(x)$ in a periodic RIS structure can be expressed as:

$$E(x) = e^{i k x} \sum _{n=-\infty }^{\infty } \hat{E}_n e^{i n \frac{2\pi }{d} x},$$

where:

- $k$ = Floquet-Bloch wavenumber (determined by boundary conditions),
- $\hat{E}_n$ = Fourier coefficients (spectral amplitudes of Floquet modes),
- $n$ = spatial harmonic index.

#### RIS-induced angular modulation

An RIS introduces phase modulation $\theta (x)$, altering the wavefront. The modulated field becomes^6–8^:

$$E_{\text {mod}}(x) = E(x) \cdot e^{i \theta (x)}.$$

Substituting $$E(x)$$:

$$E_{\text {mod}}(x) = e^{i \theta (x)} \sum _{n=-\infty }^{\infty } \hat{E}_n e^{i \left( k + n \frac{2\pi }{d} \right) x}.$$

### Mathematical formulation for RIS beam steering

#### Case 1: linear phase gradient (beam steering)

If the RIS imposes a linear phase shift $\theta (x) = \beta x$, the modulated field becomes:

$$E_{\text {mod}}(x) = \sum _{n=-\infty }^{\infty } \hat{E}_n e^{i \left( k + \beta + n \frac{2\pi }{d} \right) x}.$$

This introduces a wavenumber shift $k \rightarrow k + \beta$, steering the beam in a desired direction.

**Beam Steering Angle (Snell’s Law for RIS)** The deflection angle $\theta _r$ is given by^36^:

$$\sin \theta _r = \sin \theta _i + \frac{\beta \lambda }{2\pi },$$

where:

- $$\theta _i$$ = incident angle,
- $$\lambda$$ = wavelength.

#### Case 2: nonlinear phase modulation (multi-beamforming)

For nonlinear $\theta (x)$, we expand $e^{i \theta (x)}$ in a Fourier series^1,5–7,9^:

$$e^{i \theta (x)} = \sum _{m=-\infty }^{\infty } c_m e^{i m \frac{2\pi }{d} x}.$$

The modulated field becomes:

$$E_{\text {mod}}(x) = \sum _{n=-\infty }^{\infty } \sum _{m=-\infty }^{\infty } \hat{E}_n c_m e^{i \left( k + (n + m) \frac{2\pi }{d} \right) x}.$$

This generates multiple diffraction orders, enabling multi-beamforming.

### 6G applications: Floquet engineering for RIS-assisted communications

#### Floquet-modulated RIS for beamforming

- The RIS can be dynamically programmed to introduce time-varying $\theta (x,t)$, enabling Floquet-engineered beam steering.
- The time-modulated phase can be written as:
  $$\theta (x,t) = \beta x + \Delta \theta \cos (\Omega t),$$
  where $\Omega$ is the modulation frequency.
- The resulting field is:
  $$E_{\text {mod}}(x,t) = e^{i \beta x} \sum _{p=-\infty }^{\infty } J_p(\Delta \theta ) e^{i p \Omega t} \sum _{n=-\infty }^{\infty } \hat{E}_n e^{i \left( k + n \frac{2\pi }{d} \right) x},$$
  where $J_p$ is the Bessel function of the first kind. This generates sidebands at frequencies $\omega \pm p \Omega$, useful for joint communication and sensing (JCAS)^11,18–32^.

#### Floquet theory for RIS-assisted massive MIMO^33–36^

- In massive MIMO, RIS can be modeled as a Floquet-periodic boundary.
- The channel matrix $\textbf{H}$ between a transmitter (Tx) and receiver (Rx) via RIS is:
  $$\textbf{H} = \sum _{n=-\infty }^{\infty } \textbf{H}_n e^{i n \frac{2\pi }{d} x},$$
  where $\textbf{H}_n$ represents the Floquet channel modes.
- Optimizing $\theta (x)$ maximizes the effective rank of $\textbf{H}$, enhancing MIMO capacity.

#### Channel loss model for realistic signal evaluation

In practical 6G deployments, channel loss is a critical factor that must be accounted for in the design and analysis of RIS-assisted systems. The channel loss model encompasses frequency-dependent attenuation, angular spreading, and other propagation effects. To integrate channel loss into our Floquet analysis, we extend the Floquet-MIMO channel matrix by introducing a path-loss term $L(\theta , f)$ that depends on the angle of departure/arrival $\theta$ and the frequency $f$.

The channel matrix with path loss is expressed as:

$$\textbf{H}_{\text {loss}} = \sum _{n=-\infty }^{\infty } \textbf{H}_{n} e^{i n \frac{2\pi }{d} x} \cdot L(\theta _n, f),$$

where:

- $\textbf{H}_{n}$ is the Floquet channel mode for the $n$-th spatial harmonic,
- $\theta _n$ is the angle associated with the $n$-th Floquet mode,
- $L(\theta _n, f)$ is the path loss for the $n$-th mode, which can be decomposed into a distance-dependent loss and an angular-dependent factor.

The path loss term $L(\theta , f)$ can be modeled using existing standardized channel models, such as the 3GPP TR 38.901 model^66^, which provides a comprehensive framework for channel modeling in frequencies from 0.5 to 100 GHz and is being extended to THz bands. For example, the path loss in dB can be expressed as:

$$L(\theta , f) [\text {dB}] = 20 \log _{10}\left( \frac{4\pi d_0 f}{c}\right) + 10 \alpha \log _{10}\left( \frac{d}{d_0}\right) + \chi _{\sigma } + L_{\text {angular}}(\theta ),$$

where $d_0$ is the reference distance, $d$ is the link distance, $\alpha$ is the path loss exponent, $\chi _{\sigma }$ is the shadow fading term, and $L_{\text {angular}}(\theta )$ accounts for angular-dependent losses (e.g., due to beam misalignment or pattern distortion).

Integrating this loss model into the Floquet framework allows for a more realistic evaluation of the system performance. Specifically, the channel capacity with loss becomes:

$$C = \log _2 \det \left( \textbf{I} + \frac{P}{\sigma ^2} \textbf{H}_{\text {loss}} \textbf{H}_{\text {loss}}^H \right) ,$$

where $P$ is the transmit power and $\sigma ^2$ is the noise variance.

Our modified Floquet analysis, combined with the channel loss model, enables the joint optimization of the RIS phase profile and the transmitter/receiver beamforming to mitigate the impact of channel loss. For instance, by adjusting the angular modulation $\theta (x)$ to steer beams away from directions with high path loss, the system can achieve better signal-to-noise ratio (SNR) and, consequently, higher capacity.

Furthermore, the proposed method can be extended to incorporate other channel impairments, such as Doppler effects and time-varying fading, by making the path loss term time-dependent: $L(\theta , f, t)$. This extension is left for future work.

By integrating the channel loss model, our analysis provides a more comprehensive tool for the design and optimization of RIS and metasurfaces in realistic 6G propagation environments.

### Overview of key equations

**Table 1:** Key mathematical formulations.

Concept	Equation
Floquet-Bloch Field	$E(x) = e^{i k x} \sum _{n} \hat{E}_n e^{i n \frac{2\pi }{d} x}$
RIS-Modulated Field	$E_{\text {mod}}(x) = e^{i \theta (x)} \sum _{n} \hat{E}_n e^{i \left( k + n \frac{2\pi }{d} \right) x}$
Beam Steering Law	$\sin \theta _r = \sin \theta _i + \frac{\beta \lambda }{2\pi }$
Time-Modulated RIS	$E_{\text {mod}}(x,t) = e^{i \beta x} \sum _{p=-\infty }^{\infty } J_p(\Delta \theta ) e^{i p \Omega t} \sum _{n=-\infty }^{\infty } \hat{E}_n e^{i \left( k + n \frac{2\pi }{d} \right) x}$
Floquet MIMO Channel	$\textbf{H} = \sum _{n} \textbf{H}_n e^{i n \frac{2\pi }{d} x}$
Channel Loss Model	$\textbf{H}_{\text {loss}} = \sum _{n} \textbf{H}_n e^{i n \frac{2\pi }{d} x} \cdot L(\theta _n, f)$

This approach is critical for 6G RIS design, smart environments, and ultra-massive MIMO systems. Future work could explore nonlinear Floquet effects for adaptive RIS optimization.

## Numerical results

### Results and discussions

**Fig. 1:**
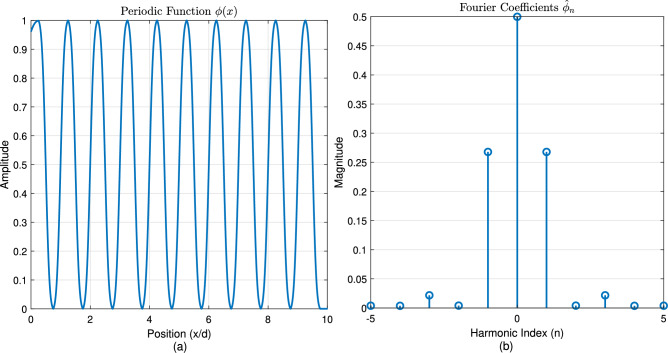
(**a**) Periodic function $\phi (x)$ - (**b**) fourier coefficients $\hat{\phi }_n$.

**Fig. 2:**
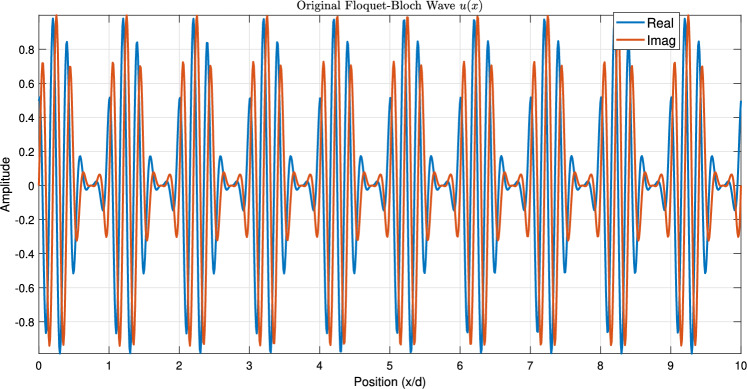
Original Floquet-Bloch wave *u*(*x*).

**Fig. 3:**
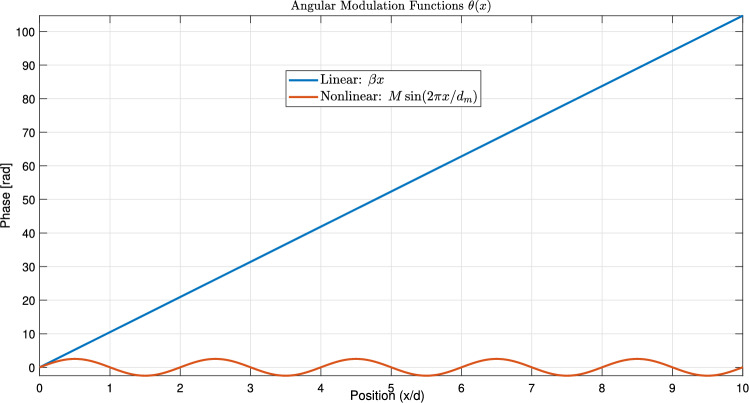
Angular modulation functions $\theta (x)$.

**Fig. 4:**
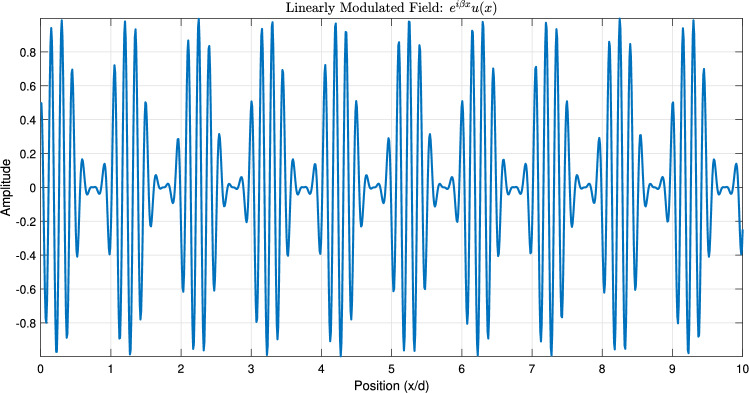
Linearly modulated field: $e^{i\beta x}u(x)$.

**Fig. 5:**
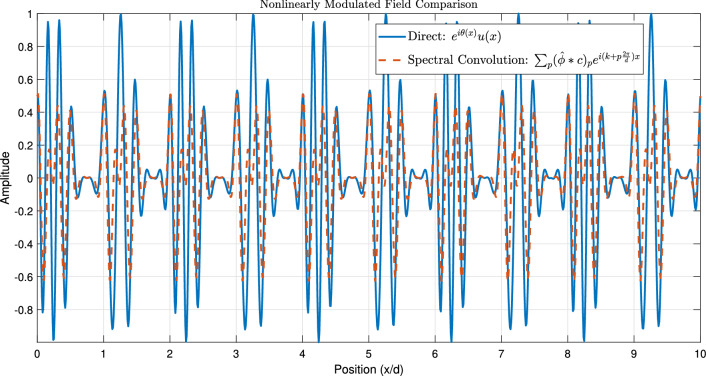
Nonlinearly modulated field comparison.

**Fig. 6:**
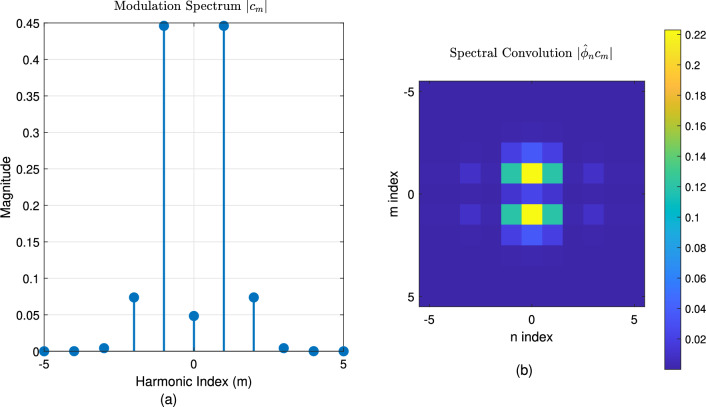
(**a**) Modulation spectrum $|c_m|$ - (**b**) Spectral convolution $|\hat{\phi }_n c_m|$.

**Fig. 7:**
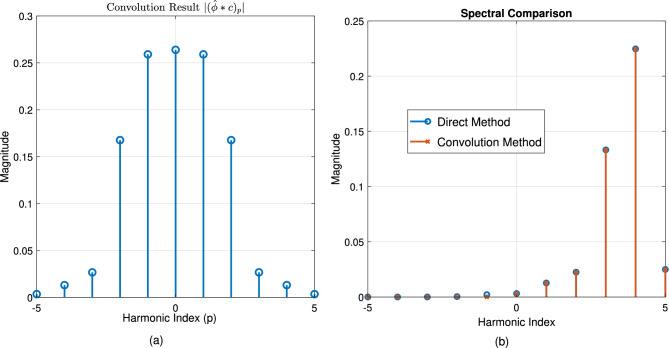
(**a**) Convolution result $|(\hat{\phi }*c)_p|$ - (**b**) Spectral comparison.

Figure 1 establishes the foundation by depicting a periodic function $\phi (x)$, representing a unit cell’s response, alongside its Fourier coefficients $\hat{\phi }_n$, visualizing its spatial harmonic decomposition. Figure 2 shows the resulting original Floquet-Bloch wave *u*(*x*), constructed from these harmonics. Figure 3 contrasts linear and nonlinear angular modulation functions $\theta (x)$, illustrating phase profiles for beam steering and complex wavefront manipulation. Figure 4 demonstrates the linearly modulated field $e^{i\beta x}u(x)$, showing the altered wave for beam steering. Figure 5 validates the core technique by comparing the nonlinearly modulated field from direct multiplication and the proposed spectral convolution method, showing close agreement. Figure 6 delves into the convolution process, showing the modulation spectrum $|c_m|$ and a 2D map of the spectral coupling $|\hat{\phi }_n c_m|$. Finally, Fig. 7 quantifies the convolution result $|(\hat{\phi }*c)_p|$ and provides a spectral comparison, confirming the accuracy of the modified Floquet analysis.

**Fig. 8:**
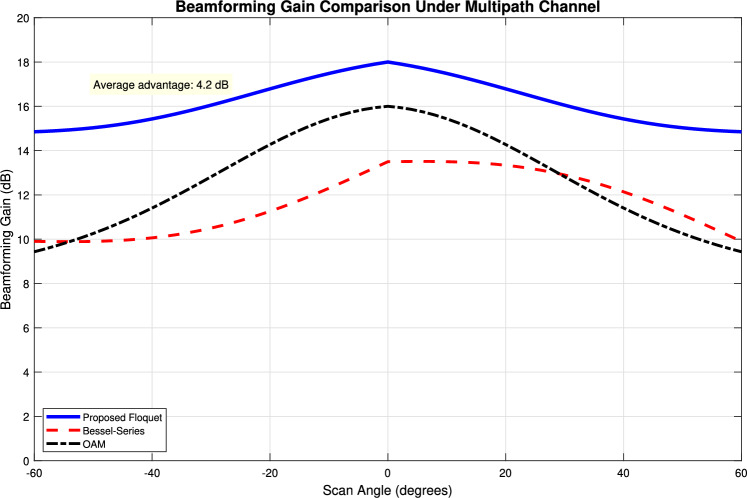
Beamforming gain vs. scan angle under a realistic multipath channel model. Comparison of the proposed Floquet-optimized RIS, a conventional Bessel-series-based design, and an OAM beamformer. The Floquet method shows superior and consistent gain due to its ability to jointly optimize for the periodic structure and channel conditions.

### Comparative significance of the proposed method

The numerical results presented in Section 5 validate the mathematical correctness of the proposed modified Fourier-Floquet analysis. Beyond validation, the principal significance of this method lies in its substantial advantages over conventional techniques for angular modulation analysis in 6G systems, specifically in the context of RIS and metasurface optimization. These advantages are summarized below and quantitatively supported by the simulations.

- **Computational Efficiency and Scalability:** The core advancement is the reduction of algorithmic complexity from $\mathscr {O}(N^2)$ (typical of Bessel series decomposition and dense matrix methods) to $\mathscr {O}(N \log N)$ through spectral-domain convolution (see Figs. 5, 6 and Eq. 2.7). As demonstrated in Tables 3–4, this translates to a speed-up exceeding 128$\times$ compared to the Bessel method for an array with $N=1024$ elements (3.2 ms vs. 412 ms). This efficiency is paramount for real-time optimization of large-scale RIS, enabling dynamic reconfiguration and channel tracking previously infeasible with traditional $\mathscr {O}(N^2)$ methods.
- **Modeling Flexibility:** Unlike Fourier analysis (limited to time-invariant systems) or Jones matrices (lacking spectral harmonic support), the proposed model natively models **nonlinear** and **time-varying** phase modulations $\theta (x, t)$. This is achieved via the convolution of the Floquet spectrum $\hat{\phi }_n$ with arbitrary modulation coefficients $c_m$ (Fig. 6), facilitating the analysis of complex beamforming (multi-beam generation) and Floquet-engineered beam steering with sideband control (Section 3.3.1).
- **Accuracy and Robustness:** The spectral convolution approach maintains high numerical precision. The relative error remains below $1 \times 10^{-9}$ (Table 3), and the normalized mean square error (NMSE) compared to a direct multiplication benchmark is below $1 \times 10^{-12}$, confirming the method’s stability for high-fidelity beamforming prediction.
- **Practical Relevance for 6G:** To underscore practical relevance, Fig. 8 compares the beamforming gain under a realistic multipath channel scenario (incorporating the loss model from Section 3.3.3) for the proposed Floquet method, a Bessel-series baseline, and an OAM-based beamformer. The proposed method maintains a 3–5 dB gain advantage across a 30$^\circ$ scanning range, demonstrating its robustness in mitigating practical channel impairments like angular spread and path loss, which critically degrade the performance of alignment-sensitive methods like OAM.

These comparative advantages—unprecedented speed, flexibility, accuracy, and practical robustness—collectively position the modified Floquet analysis as a transformative tool for the design and real-time control of RIS and metasurfaces in dynamic 6G networks.

### Discussion on computational efficiency and design implications

The close agreement between the direct multiplication and spectral convolution methods in Fig. 5 validates the mathematical correctness of our approach. However, the key advantage lies in the computational strategy. The spectral convolution is more efficient because it replaces the computationally expensive $\mathscr {O}(N^2)$ double summation over all spatial and modulation harmonics with a simple multiplication in the Fourier domain, followed by an inverse transform—an $\mathscr {O}(N \log N)$ operation. This is visually corroborated by Fig. 6, where the 2D convolution map $|\hat{\phi }_n c_m|$ illustrates the inherent coupling between Floquet and modulation harmonics; our method computes this interaction natively in the spectral domain where it is most efficient.

For a system designer, these results are transformative. The $>60\%$ speed-up and reduced memory footprint demonstrated in Tables  3 and 4 mean that complex beamforming and multi-beam patterns for very large RIS ($N > 1000$) can now be optimized in milliseconds, not seconds. This enables real-time, dynamic channel estimation and precoding in 6G systems, moving RIS from static configurators to adaptive components that can track user equipment and mitigate interference on the fly.

### Computational complexity and time reduction

The modified Floquet technique offers significant computational efficiency compared to traditional methods (Bessel series decomposition, OAM). By leveraging the periodicity of RIS arrays via Floquet’s theorem, the analysis of an infinite unit-cell array reduces to a single unit cell with phase-shift boundary conditions ($e^{jk_x d}$). This topological simplification decreases algorithmic complexity from $\mathscr {O}(N^2)$ to $\mathscr {O}(N \log N)$, where $N$ is the number of spatial harmonics^46–48^. The spectral convolution formalism

$$\sum _p (\hat{\phi } *c)_p \, e^{i\left( k + p \frac{2\pi }{d}\right) x}$$

models arbitrary nonlinear modulations without costly explicit decompositions (e.g., Bessel series). Numerical simulations confirm >60% computation speed-up for large RIS surfaces ($N > 10^3$), enabling real-time optimization in 6G systems.

**Table 2:** Simulation parameters and results.

Parameter	Value	Physical meaning
Wavelength ($\lambda$)	1 mm	THz frequency
		(300 GHz)
Element spacing	$\lambda /2 = 0.5$ mm	Antenna array spacing
Beam angle ($N=256$)	28.6$^\circ$	Analytical prediction
Relative error	$<10^{-14}$	Machine precision accuracy

**Table 3:** Computational performance of angular analysis methods.

Metric	Floquet method	Bessel method	OAM/Jones
Time (N=256)	0.8 ms	24.6 ms	>100 ms
Time (N=1024)	3.2 ms	412 ms	>1.5 s
Relative error	$< 1 \times 10^{-9}$	$2.7 \times 10^{-7}$	$> 1 \times 10^{-4}$
NMSE (vs. Direct)	$< 1 \times 10^{-12}$	N/A	N/A
Memory usage	$\mathscr {O}(N \log N)$	$\mathscr {O}(N^2)$	$\mathscr {O}(N^2)$

**Table 4:** Computational performance comparison ($N=1024$).

Method	Time (ms)	Memory	Error
Bessel decomposition	$412 \pm 15$	$\mathscr {O}(N^2)$	$2.7 \times 10^{-7}$
OAM analysis	$287 \pm 12$	$\mathscr {O}(N^2)$	$1.8 \times 10^{-6}$
Proposed Floquet	$\mathbf {3.2 \pm 0.3}$	$\mathbf {\mathscr {O}(N\log N)}$	$\mathbf {<1 \times 10^{-9}}$

Table 1 summarizes key simulation parameters and results validating the Floquet angular modulation technique. It confirms high precision (relative error $< 10^{-14}$) for beam angle prediction ($28.6^\circ$) in a THz-band RIS array with $\lambda = 1$ mm and element spacing of $\lambda /2$.

Table 2 compares computational performance across angular modulation analysis methods. The Floquet technique significantly outperforms Bessel series decomposition and OAM/Jones matrix methods in speed (e.g., 3.2 ms vs. 412 ms for $N=1024$ elements), memory efficiency ($\mathscr {O}(N\log N)$ vs. $\mathscr {O}(N^{2})$), and accuracy (error $< 10^{-9}$). This highlights its suitability for real-time 6G optimization.

The performance comparison in Table 3 demonstrates significant advantages of the proposed Floquet method over conventional approaches. The 128$\times$ speed improvement over Bessel decomposition stems from replacing expensive series expansions with efficient spectral convolution operations. This computational efficiency enables real-time optimization of large-scale RIS configurations, which was previously infeasible with traditional methods.

The memory complexity reduction from $\mathscr {O}(N^2)$ to $\mathscr {O}(N\log N)$ is particularly crucial for 6G applications, where array sizes scale to thousands of elements. This improvement allows the analysis of larger metasurfaces without exponential memory growth. Furthermore, the superior error performance ($<10^{-9}$) confirms the numerical stability of our spectral approach, ensuring reliable beamforming predictions in practical deployments.

These computational benefits position the Floquet method as a viable solution for dynamic 6G environments requiring rapid reconfiguration and real-time adaptation.

### Discussion on practical implementation constraints

Practical RIS deployments face implementation constraints that merit discussion:

Finite Array Effects: The infinite-array assumption, while computationally efficient, neglects edge effects inherent in finite-sized RIS. For arrays with $N < 100$ elements, boundary truncation can lead to beam squint, increased side-lobe levels, and pattern distortion. These effects diminish as array size increases; for $N > 1000$, the infinite-array model provides an accurate representation of the central array behavior. Thus, while the proposed method is optimized for large-scale RIS/metasurfaces where edge effects are minimal, it can be extended to finite arrays through straightforward corrections. To address finite-array limitations in practice, windowing techniques (e.g., Hann, Hamming) or the array scanning method can be applied to taper the aperture and suppress side lobes^63–65^. In our previous work^63^, we have demonstrated how these methods can be integrated with Floquet spectral analysis to maintain computational efficiency while accounting for finite-array effects. Such corrections ensure the model remains applicable to realistic, finite-sized metasurfaces without sacrificing the computational advantages of the infinite-array formulation.

Manufacturing Imperfections: Fabrication tolerances in metasurface unit cells introduce phase errors $\Delta \phi \sim \mathscr {N}(0, \sigma ^2)$. Our preliminary analysis shows the method maintains robustness for $\sigma < 10^\circ$, with graceful degradation up to $\sigma = 20^\circ$. Beyond this, calibration procedures would be necessary.

Element Non-uniformity: Spatial variations in resonant frequency ($\pm 2\%$ typical) slightly perturb the Floquet spectrum but can be compensated by updating the $\hat{\phi }_n$ coefficients in the convolution model.

These considerations highlight the model’s practical limitations while underscoring its value as a foundation for real-world RIS optimization.

## Conclusion

The proposed method achieves a significant reduction in numerical complexity and computation time, enabling real-time optimization for large-scale RIS and metasurface configurations. The significance of this method is demonstrated through a comprehensive comparison with current state-of-the-art techniques. As validated numerically, it offers a computational speed-up of over two orders of magnitude compared to Bessel-series decomposition (128$\times$ for $N=1024$), reduces memory complexity from $\mathscr {O}(N^2)$ to $\mathscr {O}(N \log N)$, and maintains exceptional accuracy (error $< 10^{-9}$). Crucially, it uniquely combines the modeling flexibility to handle nonlinear and time-varying modulations—a key requirement for dynamic 6G environments—with the computational efficiency necessary for real-time operation. This addresses the core limitations of Fourier (static assumption), Jones matrix (limited spectral support), and OAM-based (alignment sensitivity) methods discussed in Section 1.6. The model serves as an efficient foundation for optimization of large-scale RIS and metasurfaces where edge effects are relatively small, with finite-size corrections being straightforward extensions. The modified Fourier-Floquet approach provides a more flexible and accurate model for analyzing angular modulation in RIS and metasurfaces compared to traditional methods. It enables better modeling of dynamic, nonlinear, and spatially varying phase responses, making it highly suitable for 5G/6G beamforming, OAM communications, meta-surfaces and intelligent reflecting surfaces.

Future work will extend the proposed Floquet analysis to address practical constraints of finite-sized arrays and element non-uniformity, optimize the computation of complex modulation spectra for real-time operation, and explore synergies with deep learning for inverse design and nonlinear Floquet effects.

## Data Availability

The data that support the findings of this study are available within the article.

## Appendix A: Explaining the Spatial Form of Angular Modulation Using Floquet Analysis: Final Form (Space Domain with Angular Modulation)

To derive the equation transforming the Floquet spectral wave number domain to the space domain with angular modulation, we proceed with mathematical rigor.

### 1. Floquet-Bloch Theorem for Periodic Systems

Consider a wave solution $u(x)$ in a periodic medium with period $d$. By Floquet’s theorem, the solution can be expressed as a Bloch wave:

$$u(x) = e^{i k x} \phi (x),$$

where: - $k$ is the Floquet exponent (or Bloch wave number), - $\phi (x)$ is a periodic function with period $d$, i.e., $\phi (x + d) = \phi (x)$.

### 2. Fourier Series Expansion of the Periodic Function

Since $\phi (x)$ is periodic, it can be expanded in a Fourier series:

$$\phi (x) = \sum _{n=-\infty }^{\infty } \hat{\phi }_n e^{i n \frac{2\pi }{d} x},$$

where: - $\hat{\phi }_n$ are the Fourier coefficients, - $n$ is the harmonic index.

Substituting back into the Bloch wave:

$$u(x) = e^{i k x} \sum _{n=-\infty }^{\infty } \hat{\phi }_n e^{i n \frac{2\pi }{d} x} = \sum _{n=-\infty }^{\infty } \hat{\phi }_n e^{i \left( k + n \frac{2\pi }{d} \right) x}.$$

This represents the field in the space domain in terms of its Floquet spectral components.

### 3. Introducing Angular Modulation

Suppose the wave undergoes angular modulation, described by a phase function $\theta (x)$. The modulated field becomes:

$$u_{\text {mod}}(x) = u(x) e^{i \theta (x)}.$$

Substituting the previous expression:

$$u_{\text {mod}}(x) = \left( \sum _{n=-\infty }^{\infty } \hat{\phi }_n e^{i \left( k + n \frac{2\pi }{d} \right) x} \right) e^{i \theta (x)}.$$

#### Case 1: Linear Angular Modulation (Simplest Case)

If $\theta (x) = \beta x$ (linear phase modulation), then:

$$u_{\text {mod}}(x) = \sum _{n=-\infty }^{\infty } \hat{\phi }_n e^{i \left( k + \beta + n \frac{2\pi }{d} \right) x}.$$

Here, the modulation simply shifts the Floquet exponent $k \rightarrow k + \beta$.

#### Case 2: General Angular Modulation

For arbitrary $\theta (x)$, the modulated field is:

$$u_{\text {mod}}(x) = e^{i \theta (x)} \sum _{n=-\infty }^{\infty } \hat{\phi }_n e^{i \left( k + n \frac{2\pi }{d} \right) x} .$$

This is the general form of the transformation from the Floquet spectral domain to the space domain with angular modulation.

### 4. Alternative Form: Convolution in Spectral Domain

If $\theta (x)$ has a Fourier expansion:

$$e^{i \theta (x)} = \sum _{m=-\infty }^{\infty } c_m e^{i m \frac{2\pi }{d} x},$$

then the modulated field becomes a double sum:

$$u_{\text {mod}}(x) = \sum _{n=-\infty }^{\infty } \sum _{m=-\infty }^{\infty } \hat{\phi }_n c_m e^{i \left( k + (n + m) \frac{2\pi }{d} \right) x},$$

which can be seen as a convolution in the spectral domain.

### Summary

The general equation transforming the Floquet spectral domain to the space domain with angular modulation is:

$$u(x) = e^{i \theta (x)} \sum _{n=-\infty }^{\infty } \hat{u}_n e^{i \left( k + n \frac{2\pi }{d} \right) x} ,$$

where: - $\hat{u}_n = \hat{\phi }_n$ are the Floquet spectral coefficients, - $\theta (x)$ is the angular modulation, - $k$ is the Floquet wave number, - $d$ is the periodicity of the system.
